# Mastering the Best Practices: A Comprehensive Look at the European Guidelines for Cardiopulmonary Bypass in Adult Cardiac Surgery

**DOI:** 10.3390/jcdd10070296

**Published:** 2023-07-12

**Authors:** Milan Milojevic, Goran Milosevic, Aleksandar Nikolic, Masa Petrovic, Ivana Petrovic, Milovan Bojic, Sinisa Jagodic

**Affiliations:** 1 Department of Cardiac Surgery and Cardiovascular Research, Dedinje Cardiovascular Institute, 11000 Belgrade, Serbia; 2 Erasmus University Medical Center, Department of Cardiothoracic Surgery, 3015 GD Rotterdam, The Netherlands; 3 Department of Perfusion Technology, Dedinje Cardiovascular Institute, 11000 Belgrade, Serbia; 4 Department of Cardiac Surgery, Acibadem-Sistina Hospital, 1000 Skopje, North Macedonia; 5 Center of Excellence, Dedinje Cardiovascular Institute, 11000 Belgrade, Serbia; 6 Faculty of Medicine, University of Belgrade, 11000 Belgrade, Serbia

**Keywords:** guidelines, recommendations, EACTS, EACTA, EBCP, cardiopulmonary bypass, adult cardiac surgery, evidence-based medicine

## Abstract

The successful outcome of a cardiac surgery procedure is significantly dependent on the management of cardiopulmonary bypass (CPB). Even if a cardiac operation is technically well-conducted, a patient may suffer CPB-related complications that could result in severe comorbidities, reduced quality of life, or even death. However, the role of clinical perfusionists in perioperative patient care, which is critical, is often overlooked. Therefore, the European Association for Cardio-Thoracic Surgery (EACTS), the European Association of Cardiothoracic Anaesthesiology (EACTA), and the European Board of Cardiovascular Perfusion (EBCP) have agreed to develop joint clinical practice guidelines (CPGs) for CPB due to its significant impact on patient care and significant variations in practice patterns between countries. The European guidelines, based on the EACTS standardized framework for the development of CPGs, cover the entire spectrum of CPB management in adult cardiac surgery. This includes training and education of clinical perfusionists, machine hardware, disposables, preparation for initiation of CPB, a complete set of procedures during CPB to help maintain end-organ function and anticoagulation, weaning from CPB, and the gaps in evidence and future research directions. This comprehensive coverage ensures that all aspects of CPB management are addressed, providing clinicians with a standardized approach to CPB management based on the latest evidence and best practices. To ensure better integration of these evidence-based recommendations into daily practice, this review aims to provide a general understanding of guideline development and an overview of essential treatment recommendations for CPB management.

## 1. Introduction

Clinical practice guidelines (CPGs) are critical in healthcare, offering evidence-based recommendations to improve patient care quality. These CPGs are published across professional organizations and healthcare agencies at local and international levels. A multidisciplinary writing committee group, known as the Task Force (TF), comprising unpaid experts chosen for their clinical and/or academic expertise, develops these CPGs. The TF group’s roles include potential benefits and harms of treatment approaches, translating clinical and scientific knowledge into best practice recommendations, and identifying key research gaps to stimulate critical research activities in the field.

**Figure 1:**
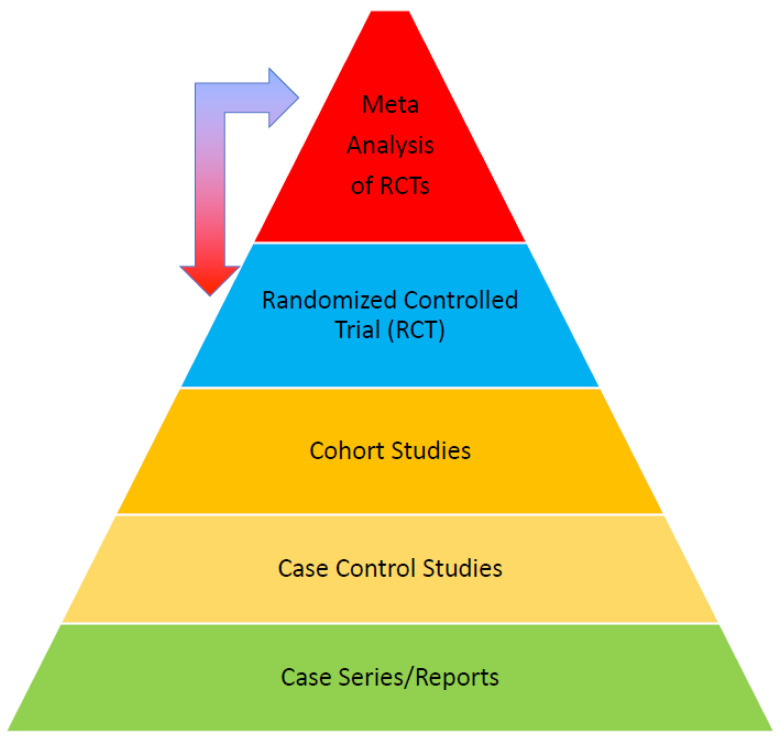
Hierarchy of evidence applied. Systematic reviews and meta-analyses of randomized controlled trials (MA-RCTs) are the most effective tools for evaluating and summarizing multiple treatment approaches when developing practice recommendations. To be useful in assessing outcomes, MA-RCTs must meet these requirements: (1) no moderate-to-severe heterogeneity, (2) moderately sized trials, and (3) a symmetrical funnel plot.

**Figure 2:**
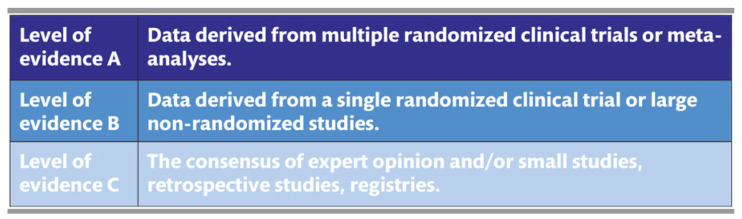
Adopted levels of evidence for clinical guidelines.

**Figure 3:**
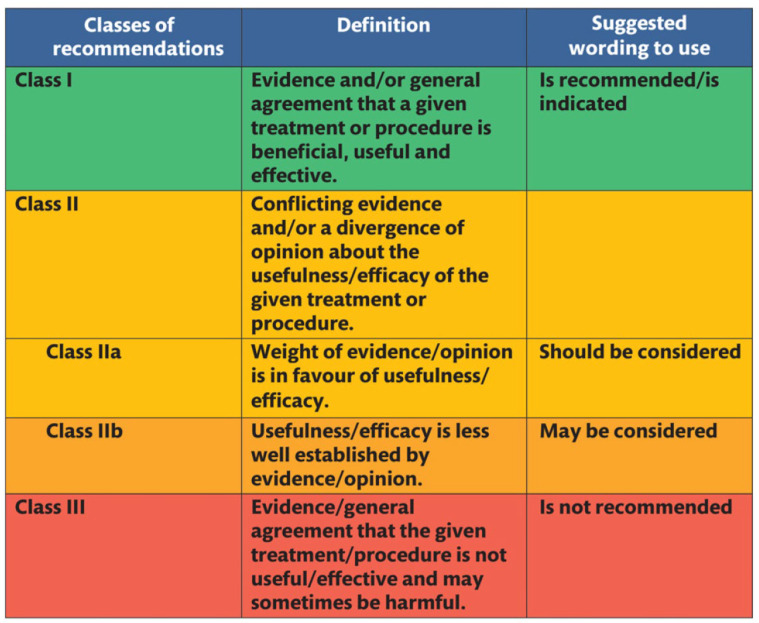
Adopted classes of recommendations for clinical guidelines.

Defined by the National Academy of Medicine (NAM) in 2011, CPGs are “statements that include recommendations intended to optimize patient care that is informed by a systematic review of the evidence and an assessment of the benefits and harms of alternative care options” [1]. They aid healthcare providers and policymakers in decision-making, supplement textbooks, standardize care, reduce variation, an improve outcomes. Guidelines are developed using a modified evidence scale hierarchy and clinical experience, with recommendations reflecting the guideline’s fundamental assertions (Figure 1). The class designation indicates the treatment’s recommendation status and the certainty around effect estimates, while the evidence level indicates the associated research strength (Figure 2 and Figure 3).

The concept of evidence level was first introduced by a Canadian Task Force in 1979, aiming to enhance population health through periodic health examinations [2]. This approach was later updated during the development of recommendations on antithrombotic agents [3]. Randomized controlled trials (RCTs) and/or meta-analyses of RCTs are seen as the most reliable evidence forms due to their less susceptible to bias and systematic errors. RCTs, being the gold standard, randomly assign participants to different treatments, minimizing the likelihood of bias. However, they can be costly and resource-demanding. In such instances, observational data such as those obtained through cohort studies or case series, could be used for assessing intervention effectiveness and harms. Despite being more prone to bias, these studies can provide invaluable data when RCTs are not feasible or ethical. Thus, the TF should review all scientific evidence, including RCTs and observational studies, to develop comprehensive guidelines reflecting the best available evidence and clinical expertise.

Guidelines encapsulate evidence on a topic to help health professionals in choosing suitable treatment options for a specific patient. Recommendations should aid in delivering patient care through the judicious use of available evidence in conjunction with clinical expertise and patient expectations. However, as guidelines cannot cover all clinical situations, the final decision remains at the discretion of the treating physician or healthcare team, considering the patient’s preferences.

Another important clinical guideline goal is to identify gaps and guide future investigations. Systematic literature reviews and group assessments help detect these gaps properly, fostering rigorous and reproducible research. By providing a framework for identifying knowledge gaps, guidelines can facilitate efficient research to resolve uncertainties about optimal diagnostic and patient care practice.

Finally, it is essential to recognize that CPGs are not without limitations. Their interpretation in real-world contexts can be influenced by factors such as the TF composition, potential intellectual biases, data insufficiency and varying result interpretations. This can lead to disparate treatment recommendations for the same clinical conditions across different TF groups [4].

Trustworthy guidelines should clearly articulate the connection between treatment options and health outcomes. They must accurately reflect the available evidence, without prematurely advancing it. Therefore, it is important that CPGs be updated every 3 to 5 years.

## 2. European Guidelines for Safe Cardiopulmonary Bypass in Adult Patients

### 2.1. Guideline Development Process

The impact of CPB makes clinical perfusion a unique surgical speciality. To address the lack of standardized practice in Europe, the European Association for Cardio-Thoracic Surgery (EACTS), the European Association of Cardiothoracic Anaesthesiology (EACTA), and the European Board of Cardiovascular Perfusion (EBCP) have formed the writing committee. The TF aimed to develop the first European evidence-based recommendations for the use of CPB in cardiac surgery, ultimately improving patient care, standardizing practices, and guiding future research in clinical perfusion.

A systematic review was conducted following strict protocols to ensure high-quality standards, as described in the EACTS Methodology Manual for Practice Guidelines [5]. The Population, Intervention, Comparison, Outcome and Timeframe (PICOT) model was used to structure clinical research questions when synthesizing evidence [6]. Literature searches were based on standardized MeSH keywords from the PubMed and Embase databases. The study endpoints assessed included hard clinical outcomes and softer, clinically meaningful outcomes such as ICU length of stay, prolonged ventilation time, bleeding complications, and allogenic blood transfusion requirements.

After assessing the quality of the reviewed studies, the risks and benefits of a particular intervention were considered to establish specific recommendations. The Task Force members reached a consensus on the final recommendations through extensive discussions and the Delphi method with a 75% voting threshold. The class of recommendations and levels of evidence were established for each statement based on the methodological quality, validity, and applicability of available clinical studies (Figure 2 and Figure 3). In the absence of proper scientific research, expert consensus statements (graded with Level of Evidence C) were used to cover specific issues of particular clinical importance for daily practice. The guidelines were reviewed by anonymous reviewers delegated by the EACTS, EACTA, and EBCP governing bodies in collaboration with the editors of official society journals [7]. These guidelines provide essential practical recommendations for healthcare professionals involved in open-heart surgery, emphasizing intraoperative patient optimization and risk reduction associated with the use of CPB.

### 2.2. Scope of the Guideline

The current guidelines summarize the scientific evidence for various recommendations covering all aspects of modern CPB practice. These aspects include (1) training, education, and service delivery; (2) machine hardware; (3) disposables; (4) preparation for initiating CPB; (5) procedures during CPB; (6) weaning from the heart–lung machine; and (7) gaps in evidence. Topics already covered in other society guidelines or expert consensus documents, such as patient blood management and post-cardiotomy extracorporeal life support in adult cardiac surgery, are briefly emphasized. Readers are redirected to these sources for more information due to strict word count limits [8,9]. Due to its complexity, the temperature management has been scheduled for subsequent work in the separate document.

### 2.3. Training, Education, and Service Delivery

A clinical perfusionist’s core function is the heart–lung machine’s operational management. Perfusionists must have adequate theoretical knowledge, skills, and experience to ensure safe and effective clinical practice [10]. Regular participation in continuing medical education and maintaining a sufficient clinical caseload are crucial for maintaining high-level competency. Effective leadership is essential for promoting continuous education and a positive work environment, ensuring a high standard of care and reducing errors. This document proposes measures and documented operating procedures to help the clinical perfusion team consistently deliver the best evidence-based patient care.

First, the perfusion department must have written standard operating procedures covering equipment use, safety measures, required competencies, continuous training, record-keeping, and responsibility for complete adherence to protocols. These procedures should be reviewed and updated annually according to the latest developments and approved by the institution. The document strongly supports the use of checklists for all CPB machine components before initiating or terminating CPB.

Furthermore, it is highly recommended that perfusionists obtain certification through a formal education period in an “approved learning training program” and complete a proper examination of skills and theoretical knowledge. Perfusionists should maintain their license by demonstrating appropriate professional development, meeting minimum caseload requirements, and participating in continued medical education (CME). These recommendations aim to prevent professional-related bias in everyday practice and keep perfusionists informed about the most recent knowledge in the field.

The perfusion department must closely monitor staffing to ensure that their organization unit’s needs are always adequately met. It is recommended that the daily number of certified perfusionists in the department be N + 1, where N represents the number of concurrently running operating rooms. For example, if there are two operating rooms scheduled for heart surgery on a given day, the department should have three perfusionists available. Since adhering to this rule can be particularly challenging outside regular working hours, an additional on-call perfusionist should be available to support personnel as needed.

Lastly, patient and personnel safety and quality improvement are crucial in all healthcare systems. Growing experience suggests several effective methods to enhance quality and safety [11,12]. Reporting and systematically analyzing errors or adverse events, including regularly presenting them for shared learning, hold absolute recommendations. Through procedural data collection of patient characteristics and outcomes, along with submitting audited variables into a hospital registry, regular data analyses should be regarded as a unique opportunity for further quality assurance and improvement initiatives. In addition to regular briefings, team meetings, and team culture self-assessment, guidelines strongly recommend institutionalizing verbal communication between team members in the operating room and ensuring it is consistently carried out in the same manner [13].

### 2.4. Heart–Lung Machine Hardware

#### 2.4.1. Console with Pumps and Holders

The console of the heart–lung machine, with holders and pumps, serves as the foundation for most disposable CPB components. However, minimal research has focused on different hardware aspects. Despite this, there is a general consensus about the safety features of CPB machines [14,15]. It is worth noting that the following heart–lung machine hardware represents the gold standard of care, even in the absence of data: pressure controllers for both arterial line and cardioplegia systems, bubble detectors, low-level sensors and alarms, electrical safety specifications, pump reversal of flow or “runaway” protection, and a written maintenance plan for the entire CPB equipment.

#### 2.4.2. Monitoring

Modern CPB management necessitates careful monitoring of the patient’s physiological and machine parameters, allowing for more complex repairs in adult heart surgery [16,17]. The document has introduced a detailed list of monitoring parameters that perfusionists can easily follow. Continuous monitoring of arterial line pressure (pre- and post-oxygenator) in the CPB circuit and continuous oxygenator arterial outlet temperature measurements are highly recommended. Additionally, it is advised to continuously monitor SvO2 and HCT levels during CPB, while blood gas analyses should be conducted at regular intervals or continuously monitored. Lastly, routinely considering the pump flow via ultrasonic measurement on the arterial line is suggested.

#### 2.4.3. Safety Features

Although the probability of fatal events associated with CPB complications has consistently decreased, numerous studies have identified potential risk factors related to specific safety cultures [18,19]. Consequently, these guidelines strongly advocate for all cardiac surgery units to record and analyze all adverse events potentially connected to CPB practice systematically and comprehensively. Efficient analysis can illuminate potential areas for improvement and facilitate the timely prevention of significant events in the future. Indeed, the online Perfusion Improvement Reporting System of the Australian and New Zealand College of Perfusionists (https://anzcp.org/pirs-ii/ accessed on 1 July 2023) serves as a valuable resource for this purpose.

#### 2.4.4. Oxygen and Air, Carbon Dioxide, and Volatile Anesthetic Supply

Volatile anesthetics are frequently employed during CPB, posing a potential risk of occupational exposure to these agents. Utilizing a scavenging system at the oxygenator outlet is the unequivocal recommendation to prevent undesirable exposure. In accordance with relevant standards, the heart–lung machine must be connected to an indexed piped medical oxygen, air, and carbon dioxide supply, with immediate backup cylinder supplies available. Possessing adequate knowledge concerning the gas supply and the effects of volatile anesthetics on oxygenators and the patient is crucial for every clinical perfusionist [20].

#### 2.4.5. Heater–Cooler Unit

*Mycobacterium chimaera* was initially predominantly associated with pulmonary infections. However, *M. chimaera* has more recently become a well-known cause of severe infections following heart surgery due to the use of contaminated heater–cooler units (HCUs) [21,22]. Several studies have demonstrated how *M. chimaera* aerosols could enter from the contaminated HCU into an open surgical field and cause infection [23]. Consequently, several preventive measures that hospitals should implement to prevent *M. chimera* contamination are recommended:

- Placing HCUs outside of operating rooms;
- Adhering to the manufacturer’s decontamination procedures;
- Establishing local safety monitoring schedules;
- Engaging in international cooperation on diagnosing, managing, and preventing dissemination to reduce the disease burden.

To reduce the burden of *M. chimaera* infection, it is essential to have a qualified laboratory conduct antimicrobial susceptibility testing of the isolates at surveillance sites on an annual basis. Proper specimen management, storage, and transportation practices are critical to ensuring accurate and reliable test results, which can then be used to inform strategies for preventing and managing infections effectively.

### 2.5. Disposables for the Conduct of Cardiopulmonary Bypass

Storage areas for disposable items must be designed to provide optimal conditions that maintain the integrity and functionality of these items. It is important to adhere to label descriptions and ensure that the designated storage space is dark, clean, and dry, protected from moisture, and maintained within appropriate temperature and humidity limits. By doing so, the quality and effectiveness of disposable items can be preserved, ensuring they are safe for use in clinical settings.

#### 2.5.1. Cannulas

Optimal outcomes in cardiac surgery necessitate effective collaboration among all team members involved in the surgical process. It is advised that the perfusionist and operating surgeon reach a preoperative consensus on the type and size of arterial and venous cannulas. This ensures safe venous return and appropriate arterial flow, customized to the patient’s procedural requirements. In cardiac surgery, potential aortic dissection or embolization due to atherosclerotic debris detaching from the aortic wall during arterial cannulation, cross-clamping, or the cannula’s sand-blast jet effect is not uncommon [24,25]. To minimize vascular embolization and decrease stroke incidence, epiaortic ultrasonography can be employed to identify ascending aorta plaques before aortic cannulation [26,27]. This technique is preferable over manual palpation for determining cannulation placement or using transesophageal echocardiography.

#### 2.5.2. Venting and Suction Devices

Unfortunately, since the introduction of cardiopulmonary bypass (CPB), there has been limited progress in understanding the cardiotomy suction (CS) mechanism. As CS remains a major source of hemolysis, it is advised to use it sparingly and avoid air entrainment into the cardiotomy and venting lines to minimize blood element traumatization [28]. This recommendation is based on two clinical studies that proposed a gentler blood aspiration technique, utilizing passive venting or an intelligent suction device for CS to decrease hemolysis rates [29,30]. However, these approaches warrant further large-scale studies before they can be endorsed as standard practice in CPB.

#### 2.5.3. Reservoirs

The choice between closed and open venous reservoirs remains a topic of debate, as both have their pros and cons, and no concrete evidence demonstrates a clear benefit of one over the other. Nevertheless, studies involving patients with low hematocrit levels (<35%) have shown reduced transfusion requirements when using closed reservoirs, likely due to the significant difference in priming volumes between the groups [31]. As a result, the panel recommends a closed venous reservoir for selected patients to lessen the inflammatory response and enhance biocompatibility. Additionally, considering a separate cardiotomy reservoir may help reduce the adverse effects of shed mediastinal blood, such as fat removal and decreased circulating leukocytes, regardless of the reservoir type [32,33].

#### 2.5.4. Oxygenators

Numerous studies have determined that membrane oxygenators outperform bubble oxygenators in terms of gaseous microemboli, complement activation, and improved neuropsychological outcomes [34]. Consequently, the TF recommend microporous membrane oxygenators as the primary choice for CPB. However, it is worth noting that polymethylpentene membrane oxygenators, which have been shown to significantly reduce volatile anesthetic concentrations, are not recommended when inhalation anesthesia is the preferred treatment for a specific surgical procedure.

#### 2.5.5. Pumps

While centrifugal blood pumps have demonstrated laboratory advantages over roller pumps, the clinical impact of a specific pump on patient outcomes remains to be determined due to inconsistent research findings [35,36]. After assessing the risks and benefits, the TF recommends using centrifugal pumps for cases with longer anticipated CPB times (CPB > 120 min). This provisional threshold highlights the need for prompt, high-quality, multicentric clinical research to address existing knowledge gaps and establish a more definitive understanding of the clinical significance.

#### 2.5.6. Filters

Although arterial line filters (ALF) are widely used in hospitals, there are limited data on their clinical benefits, especially with the reduced use of bubble oxygenators [37]. Assessing the overall clinical benefits of ALFs becomes complex when used alongside a hollow-fiber membrane oxygenator, which functions as a depth filter. The guidelines propose that ALFs could be considered for minimizing microemboli. However, as no well-designed studies have demonstrated a significant effect of leucodepletion (LD) filters on patient outcomes, the routine use of LD filters in combination with membrane oxygenators is not recommended at this time [38,39].

#### 2.5.7. Material and Surface Treatments

When blood comes into contact with the CPB circuit, it triggers a systemic inflammatory response involving leukocytes, platelets, and the coagulation system. Some studies have suggested that biocompatible coatings can help reduce this inflammatory response, as well as coagulation cascade activation during CPB and the incidence of postoperative complications [40,41]. However, before biocompatible coatings can be definitively recommended over conventional coatings, more extensive multicentric research studies are needed to confirm their benefits.

### 2.6. Preparation for Cardiopulmonary Bypass

Adequate preparation is crucial for ensuring patient safety during the CPB procedure. The NAM regards patient safety as “indistinguishable from the delivery of quality health care” and defines it as “the prevention of harm to patients” [42]. Indeed, safety is the foundation upon which all other aspects of care must be built [43].

European guidelines recommend using an institution-approved pre-CPB checklist during setup, before CPB initiation, throughout the perioperative period (weaning from CPB, post-CPB, emergent reinstitution of CPB), and during any other procedure or technique performed by clinical perfusionists. Additionally, it is advised to acknowledge the completion of the perfusion checklist during the surgical safety checklist “time out” procedure.

It is important to note that the guidelines emphasize that the effective use of checklists must be supported by additional safety features, such as multidisciplinarity, teamwork, professional communication, managerial support, and a culture of free safety and adverse incident reporting [7]. Of note, the document provides these checklists, which can be easily modified to suit an institution’s specific working environment.

Lastly, preoperative patient assessment in preparation for CPB is an essential factor that has been shown to positively impact outcomes [44,45]. The perfusionist must be well-informed about the patient’s overall condition, any coexisting diseases, the scope of the operation, and the comprehensive surgical procedure plan before initiating CPB. It is crucial that the written and/or electronic assessment document is incorporated into the patient’s medical record for proper documentation and reference [46].

### 2.7. Procedures during Cardiopulmonary Bypass

During CPB, a multitude of specific management strategies must be employed to maintain parameters approximating normal physiology, thereby ensuring optimal end-organ function, general anesthesia, pain management, and anticoagulation. Table 1 delineates the absolutely recommended and non-recommended procedures during CPB and indicates the corresponding level of evidence substantiating each statement. This section includes the addition of 25 Class IIa and 15 Class IIb recommendations for procedures or interventions that may be appropriate for the majority or individual patients. The benefits largely or moderately outweigh the given risks, but more evidence is needed before these interventions can become the standard of care. When these recommendations are applied in close collaboration with anesthesiologists and cardiac surgeons, they present a significant opportunity to enhance CPB practices and improve both short- and long-term patient outcomes.

### 2.8. Separation from Cardiopulmonary Bypass

Weaning a patient from CPB entails achieving adequate circulation, oxygenation, and organ perfusion without the need for ongoing CPB assistance. Safe and successful weaning represents a complex multidisciplinary process, in which various team members collaborate to facilitate the transition from complete mechanical circulatory and respiratory support provided by CPB to the patient’s own heart and lungs. As such, proper training, effective communication, and thorough planning among all healthcare professionals involved in the procedure are crucial during the weaning period to swiftly respond to and appropriately resolve any medical or technical issue [47]. However, conducting research and offering practical recommendations on optimal reperfusion time and strategy following ischemic cardiac arrest is challenging due to ethical concerns. Decisions regarding these matters must be made on an individual, case-by-case basis, and necessitate close collaboration among clinical perfusionists, surgeons, and anesthesiologists.

Conversely, most institutions utilize a standardized weaning protocol and/or checklist during the initial attempt to wean from CPB’s mechanical support. While no specific studies have been conducted to confirm the impact of weaning checklists on clinical outcomes in clinical perfusion, lessons learned from the anesthesia field can be extrapolated to support a strong recommendation for their routine use prior to the weaning process in order to improve performance and patient safety [47,48]. As a result, the TF has suggested a weaning checklist based on best practice standards as supplementary material, which can be promptly integrated into daily practice.

#### 2.8.1. Hemodynamic Monitoring and the Use of Positive Inotropes

A considerable amount of evidence indicates that the pulmonary artery catheter (PAC), newer minimally invasive hemodynamic monitors, and transesophageal echocardiography (TEE) can significantly contribute to successful weaning from CPB and often influence cardiac surgical decisions [49]. In accordance with the recommendations put forth by representative anesthesia societies, expert documents endorse the use of intraoperative TEE in virtually all heart and thoracic aortic surgeries, unless a clear contraindication exists [50,51]. Proper training is essential to avoid major TEE-associated complications, which are estimated to occur in less than 0.1% of cases [52]. Similarly, the use of PAC may be indicated in selected challenging cases, with the benefits and risks balanced according to a specific patient’s condition [53].

Inotropes and vasopressors are crucial treatments for reducing mortality in cardiac surgery patients with hemodynamic instability. Moreover, using phosphodiesterase inhibitors can enhance the success of weaning from heart–lung machines [54]. However, levosimendan is only conditionally recommended for hard-to-wean patients. This recommendation is based on a number of recent, well-designed studies that have failed to demonstrate any survival benefits associated with the prophylactic use of levosimendan in patients with low left ventricular ejection fraction or low cardiac output syndrome [55,56]. Consequently, it is not advised to use levosimendan prophylactically to lower the risk of complications and death. Moreover, it is not recommended to combine levosimendan with other inotropes or vasopressors [57].

#### 2.8.2. Residual Blood Management

Re-transfusing the residual volume from the heart–lung machine (CPB) circuit at the end of a procedure is a key part of blood conservation strategies. This helps minimize the need for blood transfusions from donors and their associated risks. However, the most effective method for handling the CPB circuit’s residual volume remains to be seen due to varying results from different studies [58,59]. Despite the lack of robust evidence, considering the risk–benefit ratio, the TF recommends re-transfusing the processed residual volume of the CPB circuit at the end of the procedure. This approach aims to reduce bleeding and transfusion-related complications after surgery.

## 3. Conclusions

The first-ever collaborative effort from three representative societies, involved in various interconnected aspects of patient care, has led to 113 best-practice recommendations for CPB procedures from a European perspective. Out of these, 43 (38%) are Class I, 32% Class IIa, 19% Class IIb, and 10% Class III recommendations. The Task Force also provides several practical tools such as a preoperative assessment form, monitoring parameters, a pre-CPB checklist, and a weaning checklist to assist perfusionists in their daily tasks. However, only 6% of the recommendations are backed by the highest-class Level of Evidence, A, while 50% are supported by Level B and 44% by Level C Evidence.

Knowledge gaps exist in nearly every area of the CPB management. Addressing these open questions is crucial to enhancing our collective understanding and enabling future updates to guidelines. Despite this, the current recommendations significantly strive to optimize and standardize CPB techniques worldwide. Incorporating these guidelines into hospital protocols and evaluating their impact on patient outcomes is a shared responsibility for healthcare professionals, managers, and policymakers involved in treating patients undergoing cardiac surgery with CPB. This approach leads to better patient care.

## Figures and Tables

**Table 1:** Recommended and not-recommended procedures during cardiopulmonary bypass.

Class of Recommendation: I (Recommended/Indicated: Benefit>>>Risk)	LoE ^1^	Class of Recommendation: III (Not Recommended: No Benefit or No Harm)	LoE ^1^
Carbon dioxide flush			
It is recommended that CO_2_ flush of the CPB circuit before priming be established as the standard of care to reduce GME.	**B**	No absolute recommendations exist.	
Priming volume			
Retrograde and antegrade autologous primings are recommended as part of a blood conservation strategy to reduce transfusions.	**A**	The use of modern low-molecular-weight starches in priming and non-priming solutions to reduce bleeding and transfusions is not recommended.	**C**
Anticoagulation management			
In the absence of individual heparin dosing tools, it is recommended that ACT tests be performed at regular intervals based on institutional protocols, and heparin doses have to be given accordingly.	**C**	No absolute recommendations exist.	
Mean arterial blood pressure			
It is recommended to adjust the MAP during CPB with the use of arterial vasodilators (if MAP >80 mm Hg) or vasoconstrictors (if MAP <50 mm Hg) after checking and adjusting the depth of anesthesia and assuming sufficiently targeted pump flow.	**A**	The use of vasopressors to force the MAP during CPB at values higher than 80 mm Hg is not recommended.	**B**
It is recommended that vasoplegic syndrome during CPB be treated with a1 adrenergic agonist vasopressors.	**C**	No further absolute recommendations.	
Pump flow and hemodynamic management			
It is recommended that the pump flow rate be determined before the initiation of CPB based on the BSA and the planned temperature.	**C**	No absolute recommendations exist.	
GDT is recommended to reduce the rate of postoperative complications and length of hospital stay.	**A**		
Assisted venous drainage			
It is recommended that an approved venous reservoir be used for assisted venous drainage.	**C**	Excessive negative venous pressures are not recommended due to the deleterious hemolytic effects.	**B**
It is recommended that the venous line pressure be monitored when using assisted venous drainage.	**C**	No further recommendations.	
Transfusion management			
It is recommended that PRBCs be transfused during CPB if the Hb value is <6.0 g/dl.	**C**	PRBCs should not be transfused during CPB if the HCT is >24%.	**C**
It is recommended that antithrombin concentrate to be used instead of FFP to treat antithrombin deficiency to improve heparin sensitivity.	**B**	FFP should not be used prophylactically during CPB to reduce perioperative blood loss.	**B**
Myocardial protection			
It is recommended that patient-centred myocardial protective strategies be used based on clinical condition and procedural complexity rather than on the use of a fixed institutional cardioplegic solution.	**C**	No absolute recommendations exist.	
Lung protection			
No absolute recommendations.		Leucocyte filtration and hyperoxia are not recommended for protecting the lungs during CPB.	**A**
Pharmacological management			
No absolute recommendations.		Routine use of prophylactic intravenous corticosteroids is not recommended during cardiac surgery.	**A**
Emergent institution and reinstitution of CPB			
It is recommended that a set-up CPB circuit be available at all times for emergent procedures.	**C**	No absolute recommendations exist.	
After the patient is weaned from CPB, it is recommended that the CPB circuit be kept functional until the patient’s chest has been closed.	**C**		

^1^ Level of evidence. BSA: body surface area; CO_2_: carbon dioxide; CPB: cardiopulmonary bypass; GDT: goal-directed hemodynamic therapy; GME: gaseous microemboli; FFP: fresh frozen plasma; Hb: hemoglobin; HCT: hematocrit; MAP: mean arterial pressure; PRBCs: packed red blood cells.

## Data Availability

Not applicable.
